# Developing Pandemic Prevention and Control by ANP-QFD Approach: A Case Study on Urban Furniture Design in China Communities

**DOI:** 10.3390/ijerph18052653

**Published:** 2021-03-06

**Authors:** Jing Liu, Khairul Manami Kamarudin, Yuqi Liu, Jinzhi Zou

**Affiliations:** 1 Department of Industrial Design, Faculty of Design and Architecture, Universiti Putra Malaysia, Serdang 43400, Selangor, Malaysia; gs58135@student.upm.edu.my; 2 School of Design, Kyushu University, Fukuoka 8158540, Japan; 3 School of Art and Design, Anhui University of Technology, Ma’anshan 243000, China; zoujinzhi@ahut.edu.cn

**Keywords:** urban furniture design, public health, pandemic prevention and control, Analytic Network Process (ANP), Quality Function Deployment (QFD)

## Abstract

Background: An infectious disease can affect human beings at an alarming speed in modern society, where Coronavirus Disease 2019 (COVID-19) has led to a worldwide pandemic, posing grave threats to public security and the social economies. However, as one of the closest attachments of urban dwellers, urban furniture hardly contributes to pandemic prevention and control. Methods: Given this critical challenge, this article aims to propose a feasible solution to coping with pandemic situations through urban furniture design, using an integrated method of Quality Function Deployment (QFD) and Analytic Network Process (ANP). Eight communities in China are selected as the research sites, since people working and living in these places have successful experience preventing and containing pandemics. Results: Three user requirements (URs), namely, usability and easy access, sanitation, and health and emotional pleasure, are determined. Meanwhile, seven design requirements (DRs) are identified, including contact reduction, effective disinfection, good appearance, social and cultural symbols, ergonomics, smart system and technology and sustainability. The overall priorities of URs and DRs and their inner dependencies are subsequently determined through the ANP-QFD method, comprising the House of Quality (HQQ). According to the theoretical results, we propose five design strategies for pandemic prevention and control. Conclusion: It is demonstrated that the incorporated method of ANP-QFD has applicability and effectiveness in the conceptual product design process. This article can also provide a new perspective for pandemic prevention and control in densely populated communities in terms of product design and development.

## 1. Introduction

The terminology “urban furniture” or “street furniture” emerged in the 20th century in Europe and North America, which was put forward to describe a collection of objects set up along roads, involving traffic barriers, streetlights, trash bins, bus stations, signs and post boxes, and so on [1,2]. In the early urbanization process, these objects were built to satisfy the urban populations’ fundamental requirements in public areas [3]. With the expansion of cities, urban furniture became an indispensable part of modern society and it affects most of our daily activities, being involved in accommodation, recreation, transportation, and communication [4]. People have increasing demands of urban furniture, not only for basic functions but also advanced aspects, such as safety, hygiene, comfort and entertainment, which can improve the quality of ordinary life [1,5,6,7]. However, people gain new expectations of these urban facilities under emergent circumstances, for example, a pandemic.

Since the 19th century, pathogenic viral outbreaks have resulted in complex transmission among human beings and other species, posing grave threats to public health and safety [8]. Over the past decades, humans have undergone much suffering from various pandemics, such as Spanish flu, Middle East respiratory syndrome coronavirus (MERS-CoV), hemorrhagic fever viruses (Lassa, Ebola) and severe acute respiratory syndrome coronavirus (SARS-CoV), as well as the 2009 H1N1 Pandemic (H1N1pdm09 virus) [9,10,11,12]. The most recent, COVID-19, has led to a global catastrophe that dramatically undermined social security and the economies, and we may inevitably face another pandemic in the near future [13].

Although a global pandemic is often characterized by robust infectivity and destructiveness, government agencies and policymakers cannot always implement full lockdown measures to contain it. On the one hand, pandemic situations usually last for quite a long time due to the difficulties of massive vaccination and antivirals’ development [14,15]. On the other hand, a full lockdown will lead to severe consequences, such as economic recession, unemployment and public mental disorder [16]. In this case, conditional social control measures, such as partial lockdown, travel restrictions, quarantine and social distancing, are regarded as effective treatments [17]. These social control measures change people’s behavior and lifestyles. To help people adapt to these changes and cope with pandemics, improvements in public facilities, such as urban furniture, are considered necessary.

There are three problems with the currently existing urban furniture. First, traditional urban furniture contributes little in terms of helping people adapt to a new lifestyle. For example, during the current COVID-19 pandemic, people are required to social distance from each other, but these social distancing regulations may be ignored when they are gathering around a table or in a queue. Hence, how to redesign urban furniture to provide functional assistance to people should be considered. Second, some urban furniture may become a major medium of transmission because of people’s frequent contact. Most pathogenic viruses can be transmitted by contact, including contact between humans and contact between humans and objects [18]. When people pick up expresses from express cabinets or buy soft drinks from vending machines, they touch these facilities several times. All of this contact will increase the risk of virus transmission, so urban furniture needs to be improved to reduce contact. Third, pandemic situations can also bring challenges to public mental health, but existing urban furniture does not support mental health requirements and ensure URs and DRs defined before. In this regard, how to improve urban furniture to address these issues is worth exploration.

To address problems existing in traditional urban furniture, three research questions are posed, as follows. First, what are the main user requirements (URs) of urban furniture design with respect to pandemic prevention and control? Second, what are the main design requirements DRs of urban furniture design with respect to pandemic prevention and control? Third, how can we develop design strategies of urban furniture for pandemic prevention and control based on URs and DRs? This study makes the following theoretical and practical contributions. At the conceptual level, this article contributes to the increasing body of knowledge on pandemic prevention and control. The overall priorities of URs and DRs and their inner dependencies are determined by the analytic network process–quality function deployment (ANP-QFD) method. To the best of our knowledge, this is one of the first articles exploring strategies and approaches for pandemic prevention and control in design fields. At the practical level, this study can provide references and guidance for related practical design for pandemic prevention and control.

This article is organized into the following sections: after the introduction, Section 2 presents the related literature and work. Then a decision model based on the ANP-QFD method is outlined in Section 3. A case study of urban furniture design in Chinese communities is subsequently conducted in Section 4, and the results are demonstrated. Section 5 discusses several implications of this study, while the corresponding design strategies for pandemic prevention and control are proposed in Section 6. Finally, limitations and recommendations are concluded in Section 7.

## 2. Literature Review

### 2.1. Strategies and Design for Pandemic Prevention and Control

Since the unpredictable consequences and severity of pandemics are acknowledged as inevitable, strategies aiming at pandemic prevention and control are often highly valued by policymakers and scholars. Regarding several pandemics arising in the past decades, researchers have proposed a wide range of strategies and ways of handling them. Different models and frameworks have a role to play in evaluating real situations and then assisting in finding effective solutions. For example, Degli evaluated the diffusion of pandemic influenza in Italy through an individual-based model (IBM) and put forward four effective control measures to handle the pandemic, namely, vaccination, antiviral prophylaxis, social distance and travel restrictions [19]. Halloran utilized different models to simulate a pandemic outbreak in a city with a population of 8.8 million, similar to Chicago, USA, finding that, with the transmissibility of viruses, household precautions were also necessary implements for containing the pandemic [20]. Similarly, Wu’s research model demonstrated that the integration of quarantine, self-isolation and antiviral therapy could mitigate and control pandemic situations [21].

On the other hand, practical achievements on pandemic prevention and control can fall into three primary contexts: transportation protection, inspection and diagnosis, and disinfection. In terms of transportation protection, Phoenix Air developed the Aeromedical Biological Containment System (ABCS) to provide support to researchers who surveyed bird flu in East Asia, meanwhile playing an essential role in finding infected patients in West Africa during the Ebola situation [22]. Similarly, the FAI Aviation Group, Germany’s largest business jet fleet operator, designed a portable isolation device called the EPI-Shuttle, which can prevent transmission among patients and health workers during the delivery process [23]. Regarding inspection and diagnosis, a quick detection device focusing on tuberculosis was invented by Fujifilm, assisting physicians in diagnosing tuberculosis in an early stage. To cope with the COVID-19 pandemic, Chinese companies designed a body-temperature monitor system and applied it to the filtering of groups with fever symptoms [24]. Similarly, high-performance infrared thermal cameras were set up in airports in Taiwan for quick inspection [25]. Concerning disinfection, smart disinfection robots integrating multiple disinfection methods were created to kill viruses in public facilities. These empirical studies and practices have contributed a great deal to the knowledge of prevention and control during pandemic situations.

In addition to product schemes, various service measures have been explored by scholars across the world during pandemics. For instance, Liese developed a novel online service program named Smartline, providing social support for many individuals struggling with substance use disorders (SUDs) in communities during COVID-19 [26]. Zaidi conducted a national survey to provide pharmacy delivery services for community pharmacists across the UK [27]. Similarly, Lim’s study tried to help community pharmacy teams offer medication services to groups with dementia [28]. In the USA and Canada, Wendt identified real challenges for in-clinic treatment during the pandemic and attempted to introduce expanded telemedicine services into communities [29]. In Singapore, researchers in the pediatric healthcare field proposed several innovative service practices for patients, such as teleconsultation, IT facilities, customized staff training and parent education [30].

### 2.2. Urban Furniture

Currently, the development of urban furniture tends to follow two main characteristics: one is diversity, while the other is intellectualization [31]. Urban furniture differs from region to region due to the variety of demographic, economic, cultural and lifestyle factors [32]. In China, dozens of electrical charging piles support charging services in communities, since many people are used to riding electric bicycles for their daily commute. However, few electrical charging piles are observed in communities in some South Asian countries, where the motorcycle is the dominant choice for transportation. In modern society, urban furniture embedded with a smart system that accesses valuable contextual information or services is a mainstream design, dating back to recent decades [33]. An example from an earlier period is a self-service kiosk called the Plato Hotline System, developed in 1977, providing detailed information of a university for students and school staff [32]. After that, urban furniture combined with new technologies formed the following research trend. In 1987, the Apple Company designed an electronic kiosk to gather data in shared spaces, serving as a unique platform to obtain URs [34]. The subsequent development of urban furniture has followed a more emotional and humanized route since the 21st century. In 2012, a new proposal for vulnerable groups or the disabled was launched, indicating that emotional and accessible elements would attract increasing attention [35]. Currently, urban furniture incorporating smart technology is mainstream in urban management. For example, urban furniture with smart systems have been employed as a new platform for information exchange between cities and urban settlements in smart city situations [31]. Moreover, smart urban furniture with sensors can collect various data describing air quality, traffic and noise of cities, providing useful feedback on city dynamics for policymakers [36]. A more recent study also shows that an urban light system with virtual reality technology could effectively provide an immersive experience for local citizens [37].

### 2.3. QFD

**Figure 1:**
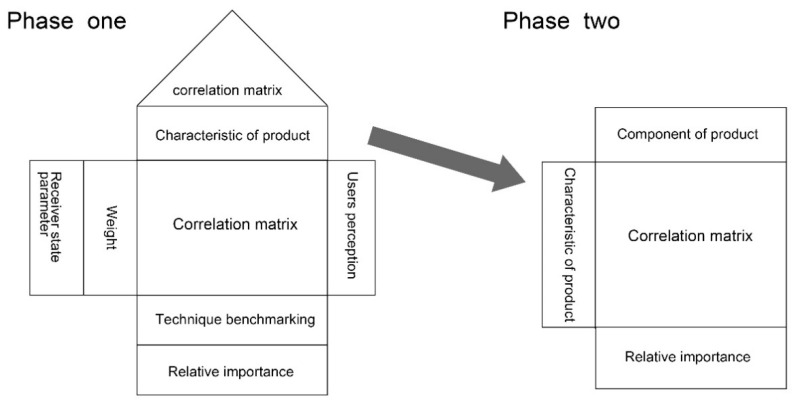
Scheme of quality function deployment (QFD) for product design (adapted from [41]).

Quality function deployment (QFD) is a scientific approach developed in Japan in 1966, transforming URs into engineering characteristics during the product development stage [38]. This method can be employed for multi-level deductible analysis in a quantity management system and help enterprises plan their products and services. The primary tool for QFD is the House of Quality (HQQ) that can identify and classify URs (what) and DRs (how) [39]. Moreover, this method can demonstrate associations between these two types of requirement, and then assign objectives and priorities for them. The scheme of QFD for product design is depicted as follows, see Figure 1 [40]:

- Receiver state parameter (left side): The receiver state parameter represents the facets of a product system, which have some kind of influence on the product system receiver. In other words, it describes the value of the receiver offered by the product system solution. In the product design context, the receiver state parameter is often represented by user expectations or URs of the product system. In this article, it represents the requirements of different stakeholders in urban furniture design for pandemic prevention and control, including government agencies, community workers, urban dwellers and health workers;
- Characteristics of product (top): This part reflects the corresponding elements of a product that can characterize the product system. In this article, this denotes the DRs of urban furniture for pandemic prevention and control;
- Component of product (top): The product component represents the product characteristics that the whole product system can be divided into. Notably, in this article, urban furniture is regarded as an entire product rather than several product components, so phase two in the Scheme of QFD in phase two (for the component of products) will not be considered.

### 2.4. ANP

The Analytic Network Process (ANP) is a multi-criteria theory of measurement employed to prioritize absolute values from individual assessments, which is a more general form of calculation compared to the Analytic Hierarchy Process (AHP) [42]. ANP has been proven to be a more effective tool than AHP when calculating the significance of several different attributes, as this method can further shed light on internal dependencies and relations among these attributes. The basic steps for using ANP are described as follows: (1) Construct a set of hierarchical network models, aiming at a specific problem, is constructed. (2) Make pairwise comparisons based on data sets. (3) Build a supermatrix to determine the overall priorities of all elements and their inner dependencies at each level (4) Make decisions based on results. The ANP method primarily contains three types of matrix: supermatrix, weighted supermatrix and limit matrix.

Scholars have conducted many practical applications of ANP in their studies. For instance, Dano employed this method to assess flood susceptibility in Malaysia and manage its impact on people and the environment [43]. Giannakis highlighted the potential dependencies among several sustainability performance indicators by using ANP [44]. However, some researchers hold the view that the traditional ANP method may have some demerits, such as uncertainty and absolute subjectivity, so they propose refined methods to overcome this shortage. Some add fuzzy variables in their studies to overcome the inaccuracy of the traditional ANP method. For example, Hemmati developed a Fuzzy ANP model for maintenance selection in an acid-manufacturing company [45]. Alilou prioritized watershed health factors to manage watersheds more efficiently by using the Fuzzy ANP [46]. Others believe that combined approaches are more likely to develop the strength of the ANP method. For instance, Parkouhi employed a mixed model combining ANP and the grey VIKOR technique to measure the resiliency of the supply chain [47].

### 2.5. The Integrated ANP-QFD Approach

**Figure 2:**
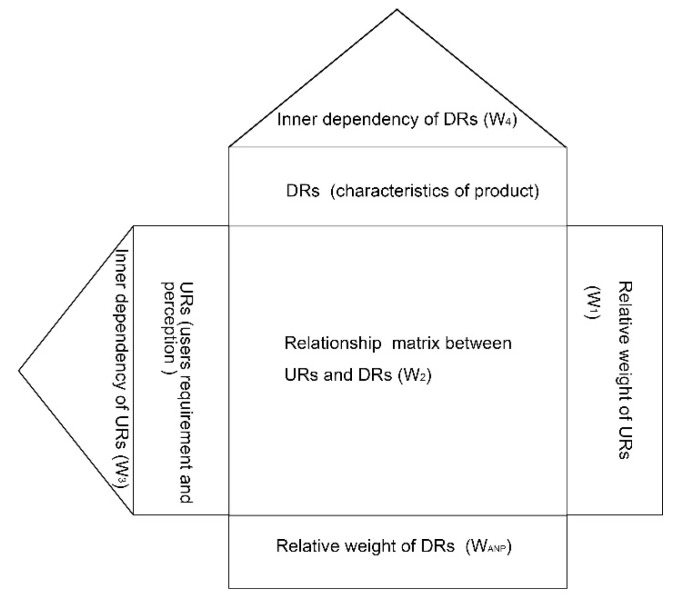
The integrated ANP-QFD method for product design (adapted from [41]).

We chose the QFD method as one of the main tools in this article, since its application has been proven useful in converting the “voice” of customers into technical requirements, which has proved to be effective in many fields, such as green supplier selection, service quality evaluation for public transportation, decision making and supply-chain operation management [48,49,50,51]. Regarding product design and development, QFD allows researchers to support engineers in the suitable identification, evaluation and characterization of URs [52]. In the service design area, Chowdhury forwarded a multi-phased approach based on QFD and first discussed the concept of “sustainable service design” [53]. Although QFD is a popular tool used to deal with URs and DRs in the product design context, this method has its limitations. Usually, we cannot determine URs’ relative importance through a single QFD approach when conducting design-planning or decision-making, which may cause inaccurate justifications for important URs and DRs. In this case, QFD is usually integrated with other research methods to overcome its shortages, such as the Kano Model, AHP, Fuzzy logic sets and ANP. As illustrated above, the ANP method can determine the relative weight of the elements of a product and their inner dependencies, facilitating a variety of interactions and feedback between tangible or intangible factors [54]. Therefore, we attempt to use an integrated ANP-QFD approach is employed in our study to explore the URs and DRs of urban furniture design for pandemic prevention and control in our study. When combined with ANP, the scheme of QFD for product design will be improved as Figure 2, where:

- W_1_ is an eigenvector that demonstrates the significance degree of each UR;
- W_2_ is a correlation matrix that demonstrates the pairwise comparison results for each DR with respect to each UR. In other words, it presents the relationships between URs and DRs;
- W_3_ is a correlation matrix of URs that demonstrates the pairwise comparison results. In other words, it presents the inner dependencies of URs;
- W_4_ is a correlation matrix of DRs that demonstrates the results of the pairwise comparison for each DR with respect to each DR. In other words, it presents the inner dependencies of DRs;
- W_ANP_ is an eigenvector that demonstrates the importance level (the relative weight) of DRs.

In the improved scheme, the relative weight of URs and DRs and their inner dependencies are calculated by the ANP method, providing a more in-depth understanding for the decision-makers deploying the resultant in the product design context. The integrated ANP-QFD can address the uncertainty, vagueness and inaccuracy of traditional QFD, which has been proved to be effective in previous studies [55,56,57]. In the product design context, many researchers have also regarded the integrated ANP-QFD as a useful tool. For instance, Geng put forward a three-domain conceptual product design framework, including user-, functional- and product-service domains [58]. Fargnoli pointed out that the integrated ANP-QFD allows engineers to find the factors that indirectly impact user satisfaction [59]. Zaim argues that using the integrated QFD-ANP method can provide sufficient quantitative precision to classify product characteristics, thereby fulfilling users’ expectations [60]. From these studies, it can be seen that the drawbacks of traditional QFD can be solved by combining it with ANP, and a more accurate evaluation and assessment of product factors can be made. However, the number of such studies is relatively low.

### 2.6. Research Gap

After reviewing the previous literature, we can find the following research gaps. First, several practical products have been developed for pandemic prevention and control, but there is a lack of systematic and theoretical exploration. Second, although a variety of service measures, such as health services, delivery services and online training services, coping with pandemics in communities, have been investigated by scholars for pandemic prevention and control, few of them draw attention to the improved design of existing products, especially for those close attachments in our daily life. Third, the ANP-QFD approach that can map URs with DRs is never applied to urban furniture design or pandemic prevention and control. Therefore, it is crucial to comprehensively address these barriers and gaps comprehensively.

## 3. Methods

**Figure 3:**
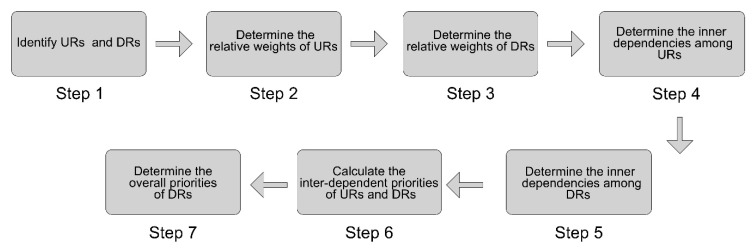
Research steps.

This research work aims to cope with the problems existing in traditional urban furniture during pandemic situations. Three research questions are proposed to identify URs and DRs in urban furniture design for pandemic prevention and control, thereby understanding how to develop effective design strategies based on theoretical results. The integrated ANP-QFD method is expected to show advantages in dealing with the three research questions that we put forward with respect to urban furniture design. The research steps are listed as follows; see Figure 3:

- Step 1. Identify the URs and DRs of urban furniture design with respect to pandemic prevention and control;
- Step 2. Determine the relative weight of URs through pairwise comparisons. There is an assumption that no UR has a dependency on another in this stage. A 9-level Likert scale is employed to make a pairwise comparison, where level 9 represents the most significant degree of the matrix while level 1 represents the least significant degree of the matrix. Once a pairwise comparison is conducted, the consistency of results will be checked immediately. Finally, the relative weights of URs will be calculated, which is described as eigenvector W_1_;
- Step 3. Determine the relative weight of DRs with respect to each UR by pairwise comparisons. Similarly, we assume that there is no inner dependency among DRs. When the pairwise comparison is completed, whether the result is consistent will be checked. The results of this step are described as eigenvector W_2_;
- Step 4. Determine the inner dependencies among URs with respect to each UR. In this step, we employ the same calculation process as in step 2. The results will be represented by W_3_. It is worth noting that URs may be dependent on each other while pairwise comparisons are processing;
- Step 5. Determine the inner dependencies among DRs with respect to each DR by using a similar calculation process to that in step 3. There may be some inner dependencies among DRs. The final results are represented by W_4_;
- Step 6. Calculate the interdependent priorities of URs by employing Formula (1): W_C_ = W_3_*W_1_. Simultaneously, calculate the interdependent priorities of DRs by utilizing Formula (2): W_A_ = W_4_*W_2_;
- Step 7. Determine the overall priorities of DRs by employing Formula (3): W_ANP_ = W_A_*W_C_.

## 4. A Case Study: Urban Furniture Design for Pandemic Prevention and Control in Chinese Communities

### 4.1. Research Design

**Figure 4:**
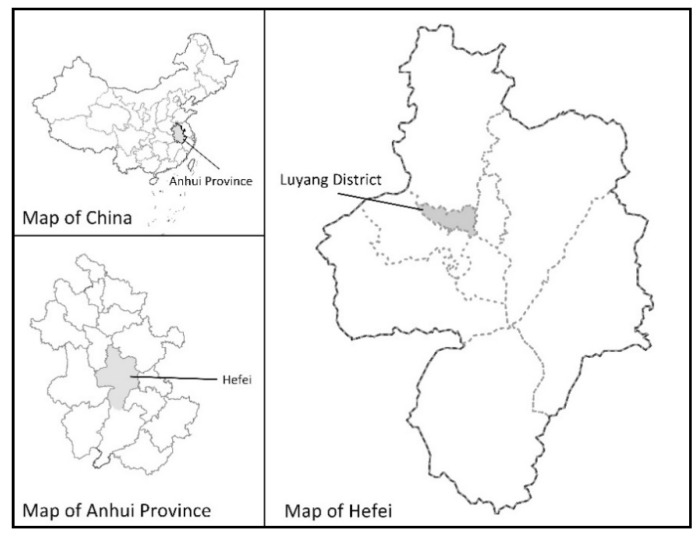
The position of Luyang District.

**Figure 5:**
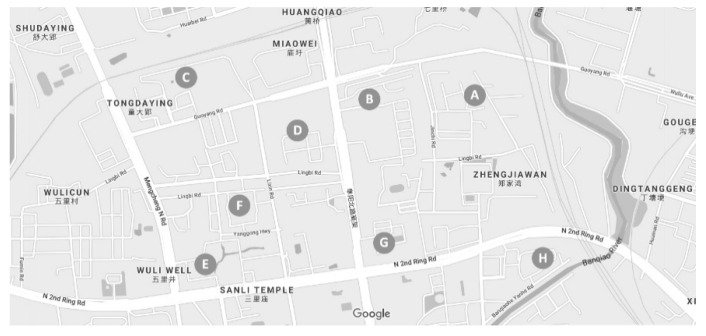
The distribution of eight selected communities on Xinglin Street.

In our research, a case study of urban furniture design for pandemic prevention and control was conducted to identify the effectiveness of the integrated ANP-QFD approach. Several common kinds of urban furniture in Chinese communities are chosen for a survey, including express cabinets, fitness facilities, charging piles, seats, streetlights, trash bins, signs and vending machines (see Table 1). Regarding the research scope, we selected eight communities on Xinglin Street in Luyang District, Hefei. According to the government report, Xinglin Street is located in the northern part of the city center, occupying an area of 3.23 square kilometers, with a total of 60 thousand people living there. We chose these Chinese communities as the research scope since people working and living in these places have a successful experience in pandemic prevention and control, particularly for the COVID-19 pandemic. On the other hand, the densely populated living conditions will increase the difficulty of pandemic prevention and control, so the experience is worthy of exploration. The detailed locations of our research sites are shown in Figure 4 and Figure 5.

Our data collection process contained two phases. The first phase determined the URs and DRs of urban furniture design for pandemic prevention and control. During this phase, a user interview was conducted. During the user interview, a total of twenty-two respondents from diverse backgrounds were chosen to participate in our study, involving seven community workers, three community pharmacists, two PhD students in community development, two industrial designers, three university teachers in design, three PhD students in design and two technicians. Their information is depicted as follows, see Table 2. Since some of the informants working in the communities were not far from us, we conducted a face-to-face interview. For the remaining informants, we conducted an online interview, mainly through two social media applications, namely QQ and We-chat, due to the far distance. After that, we integrated the results of the user interview with a literature review to obtain the final DRs and URs of urban furniture design. The second phase is for pairwise comparisons of URs and DRs. During the second phase, a second user interview was conducted, as well as a questionnaire survey. Notably, the informants in the two user interviews were the same. Regarding the questionnaire survey, 200 questionnaires were distributed to community workers, community managers, and local residents in these eight communities, including half of the online questionnaires and half of the paper questionnaires. Ultimately, we received 164 valid answers, which were used for data analysis. The demographic information and partial contents of the questionnaire survey respondents are demonstrated in Table 3.

### 4.2. Research Steps

#### 4.2.1. Step 1

In step 1, a user interview and literature review were conducted to obtain URs and DRs. Three URs are identified for further analysis; see Table 4. Among these URs, usability and easy access (UR 1) was regarded as a significant user requirement by many inhabitants for pandemic prevention and control, aiming to provide safe, effective and efficient using conditions for users. This represents the features that the user wishes to approach easily and good layout, directly impacting user-perceived quality and indirectly affecting user’s desire to return [61]. Second, sanitation and health (UR2) can also influence users’ satisfaction and expectations in urban furniture design, which has been verified in the previous literature. Lee found sanitation and health to relate positively to perceived service quality, and this finding is consistent with Vilnai-Yavetz’s research [62,63]. Particularly during pandemic situations, most respondents pay closer attention to this requirement, since they will have a stronger sense of security if this requirement can be fulfilled. Third, emotional pleasure (UR 3) was posed by many respondents in our research. As Dr. Norman described in his book, each individual has emotional requirements to fulfill and emotion plays a significant role in their understanding of the world and how they process information and make decisions [64]. Urban furniture is considered as an object that transforms urban spaces into livable spaces by adding emotional comfort [32]. However, it is worth noting that urban furniture should also assume responsibility in easing people’s moods during pandemic contexts, such as decreasing sadness, disappointment, anxiety, and confusion, and help them cope with stress. In our study, we focus on how to mitigate people’s negative feelings during pandemics through urban furniture design.

Seven DRs for urban furniture design were determined, as listed in Table 5. First, contact transmission is a major transmission method for many viruses, such as the most recent COVID-19. Urban furniture, closely involved in our daily life, is expected to lower the risk of contact transmission through improved design. In this article, contact reduction (DR1) contains two aspects: one aspect is designed to reduce contact between humans, which is also called maintaining social distancing. The other aspect is designed to minimize contact between humans and objects. This requirement has a detailed explanation in the document launched by the Chinese Center for Disease Control Prevention (CDC) in 2020. As the document stated, people should social distance (usually one meter) from each other when outside [65]. Second, during pandemics, all infrastructures and facilities in public spaces are required to undergo effective disinfection (DR2), in order to reduce the probability of transmitting viruses [65]. In this regard, urban furniture and some public facilities should be redesigned to have a disinfection function. For example, currently, security inspection devices in subway stations are designed to sterilize the packages in Hongkong, and this has proven to be effective in containing COVID-19 pandemic. Third, in the product design context, users’ emotional requirements can be responded to by products’ appearance (DR3). In other words, products with good appearances (DR3) can have emotional resonance to users. According to Dr. Norman’s research, aesthetically pleasing products appear more effective to the user, since the user can experience affinity with a product that appeals to them, as well as an emotional relationship with products [64]. This design requirement has also been discussed in Haris’s research, where appearance and furnishings are a determinant of loyalty [66]. In this article, we will concentrate more on how to ease people’s negative emotions through the appearance and design of urban furniture. Fourth, in modern society, urban furniture often assumes the responsibility of representing a city’s own distinct culture, giving city-dwellers a sense of cultural identity and belonging. This is a description of the social and cultural symbol (DR4). Urban furniture often determines a city’s initial impression, somehow expressing what a city wants to tell citizens. When traveling to a new city, the local urban furniture can show what the city wants to tell you, in terms of culture, social context and history. In other words, the image of local urban furniture is the image of the city, which explains why cultural elements are often integrated with urban furniture design. In many cities, the design of urban furniture contributes sufficiently to a city’s identity and develops a city’s image [32]. Fifth, ergonomics (UR5), is concerned with usability and availability, involving a fluent operation procedure, immediate feedback and a clear target [67]. Sixth, with the rapid development of the Internet of Things (IoT), public facilities and infrastructure equipped with smart systems and technology (DR6), such as environmental sensors, wireless modules, processors, and microcontrollers, are becoming mainstream. These advanced technologies can be useful in pandemic prevention and control. For example, a smart temperature monitor system can filter groups with an abnormal temperature, which effectively avoids disease transmission [68]. Similarly, smart wearable devices can be used to gather a healthy status from patients, hence preventing virus transmission among patients and healthcare workers. The seventh factor, sustainability (DR7), generally refers to saving energy and resources, reducing pollution and protecting the environment through design [69]. Notably, some respondents suggested that we should also focus on urban furniture design’s continuous function for pandemic prevention and control. As we know, each pandemic situation can only last for a certain period, and humans will ultimately contain the pandemic through mass vaccination and the employment of antiviral drugs. In this regard, how to prolong the role and function of urban furniture by ensuring it can help deal with the next pandemic or common infectious diseases is an important aspect of sustainability we should consider.

#### 4.2.2. Step 2

In this step, the relative importance of URs was identified through pairwise comparisons (calculation of W_1_), assuming that there was no inner dependency among URs. The respondents were asked what the relative weight of UR1 was, compared with UR2, in terms of pandemic prevention and control. A 9-level Likert table was employed for inquiry (see Table 6), where level 1 indicates that one matrix is equivalently important as to the other matrix in terms of importance. In contrast, level 9 means that one matrix is dramatically more important than the other matrix. After collecting the pairwise comparison data, the “SPSSAU”, an online statistical software, was employed to calculate the relative weights of URs. The relative weights of URs are listed in Table 7, where sanitation and health (UR2) has the highest weight of 0.589, while usability and easy access (UR1) receives the second highest weight of 0.252. The consistency ratios of pairwise comparisons were subsequently examined to assessthe validity of the data.

#### 4.2.3. Step 3

The relative weights of DRs were determined with respect to each UR (calculation of W2). For example, respondents were asked “what is the relative weight of contact reduction (DR1) when compared with effective disinfection (DR2) in terms of achieving usability and easy access (UR1)”. Then, informants were asked similar questions while varying the DRs that are the object of comparison. The relative ratios of DRs regarding usability and easy access (UR1) are shown in Table 8. Thereafter, the same method was employed to determine the relative weights of DRs regarding the remaining three URs, and the results of pairwise comparison are shown in the Table A1 and Table A2. Similarly, the relative ratios of DRs in terms of the other two URs can be identified; see Table 9. It can be derived that ergonomics (DR 5) has the highest weight of 0.334 regarding usability and easy access (UR1), while smart system and technology (DR6) shows the strongest associations with sanitation and health (UR2), with a weight of 0.327.

#### 4.2.4. Step 4

In this step, the inner dependencies of URs were identified through pairwise comparisons (calculation of W_3_). The question was “what is the relative significance of one UR compared with the other with respect to achieving UR1, UR2 and UR3, respectively”. URs which did not influence others would be excluded. The relative weights of URs with respect to each other UR are shown in Table 10, while the respective pairwise comparison results can be found in the Table A3, Table A4 and Table A5.

#### 4.2.5. Step 5

The inner dependencies among DRs were computed through the method mentioned above (calculation of W_4_). Similar to the process in the last step, respondents wererequired to answer the question: “What is the relative significance of one UR compared to the other to achieve DR1 to DR7, respectively”. A similar approach was employed to generate the results of pairwise comparisons for the remaining DRs. Table 11 demonstrates the overall inner dependencies of DRs, from which it can be seen that DR6 has more influence on DR1 than other DRs in achieving DR1. It is worth noting that DRs with no impact on others are signified with the number 0. For instance, DR3 does not have any impact on DR2 in achieving DR2.

#### 4.2.6. Step 6

After that, the inter-dependent priorities of URs would be calculated by using Formula (1). Regarding the W_C_ of UR1, the value can be calculated as W = 0.681*0.252 + 0.284*0.589 + 0.123*0.159 = 0.358. Similarly, the remaining values of W_C_ can be computed, hence

(1) $$W_{C}=\left[\begin{array}{c} 0.358\\ 0.466\\ 0.176 \end{array}\right]$$

On the other hand, using Formula (2) (W_A_ = W_4_*W_2_), the inter-dependent priorities of DRs can be determined, while

(2) $$W_{A}=\left[\begin{array}{ccc} 0.083 & 0.156 & 0.041\\ 0.222 & 0.195 & 0.062\\ 0.044 & 0.043 & 0.242\\ 0.036 & 0.036 & 0.178\\ 0.202 & 0.170 & 0.076\\ 0.348 & 0.348 & 0.179\\ 0.065 & 0.052 & 0.222 \end{array}\right]$$

#### 4.2.7. Step 7

Finally, based on the values of W_C_ and W_A_ obtained in the previous steps, the overall priorities of DRs can be acquired through Formula (3) (W_ANP_ = W_A_ * W_C_), and the resultant of W_ANP_ is

(3) $$W_{\mathrm{ANP}}=\left[\begin{array}{c} 0.110\\ 0.181\\ 0.078\\ 0.061\\ 0.165\\ 0.318\\ 0.087 \end{array}\right]\;=\;\left[\begin{array}{c} DR1\\ DR2\\ DR3\\ DR4\\ DR5\\ DR6\\ DR7 \end{array}\right]$$

**Figure 6:**
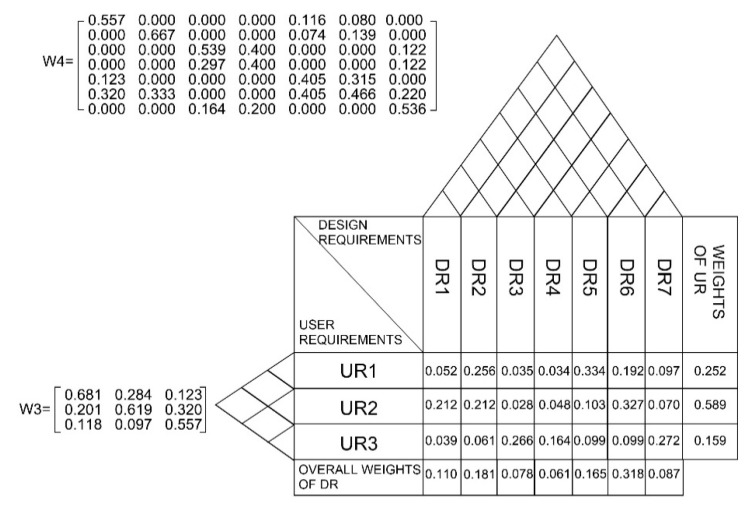
House of quality (HQQ).

According to W_ANP_, the most crucial DR is smart system and technology (DR6), with a relative weight of 0.318. Notably, effective disinfection (DR2) and ergonomics (DR5) have values of 0.181 and 0.165, respectively, which are the second and third most important DRs. Contact reduction (DR1) receives a relative weight of 0.110, which is also significant as well. Besides, a “house of quality” (HQQ) is constructed based on traditional QFD, which presents the associations among URs and DRs. The HQQ presents links between URs and DRs from different perspectives, in which the relative weight of URs and DRs, and their inner dependencies, are illustrated. The overall priorities of URs and DRs are shown in the HQQ (see Figure 6).

### 4.3. The Validation of the Integrated ANP-QFD

**Figure 7:**
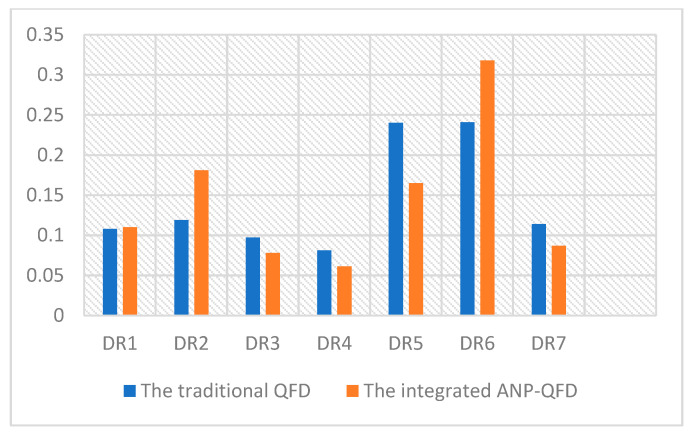
Relative weights of DRs in the traditional QFD and the integrated ANP-QFD

To better appreciate the merits deriving from the ANP-QFD approach and verify the effectiveness of the decision model, an additional analysis process is conducted. During this process, informants are asked to evaluate the relative weights of respective URs and DRs based on the traditional QFD method. Like the previous section, questionnaires are distributed to obtain the relative importance and ranking of DRs. To make an exact comparison between the integrated ANP-QFD and the traditional QFD method, the relative weights and rankings for each DR in terms of the two methods are demonstrated in Table 12 and Figure 7. Notably, as the number of URs is relatively lower, we only show the comparison results of DRs.

As illustrated in Table 12 and Figure 7, there is an evident difference between traditional QFD and the integrated ANP-QFD. On the one hand, the rankings of DRs are different in these two approaches. Table 12 demonstrates the rankings of DR1, DR2, DR5 and DR7 experience a change from the traditional QFD to the integrated ANP-QFD. It is because the integrated ANP-QFD allows more apparent evaluations among DRs, compared with the traditional QFD [66]. On the other hand, there are more distinctions of DRs in terms of the relative weights in the two approaches. For example, DR5 and DR6 have almost the same high weight in the traditional QFD. Conversely, their weights are different in the integrated ANP-QFD. Similarly, regarding the remaining matrix, the relative weights of them are also not clear and differential in the traditional QFD. As Figure 7 shown, DR1, DR2, DR3 and DR7 almost have the same weight around 0.1 in the traditional QFD, while this result is much more distinctive in the integrated ANP-QFD. According to the previous literature, the differences in matrix weights are not evident since the traditional QFD does not consider the inner relationships between URs and DRs [70]. Hence, it can be derived that the integrated ANP-QFD shows advantages in providing an objective elicitation of users’ expectations and valuable information of product characteristics.

## 5. Discussion

To respond to the heavy threats imposed by pandemic situations, we attempt to improve the currently existing urban furniture, using a systematic method that integrates ANP with QFD. We prove the effectiveness of the integrated ANP-QFD approach in dealing with URs and DRs and their inner dependencies in the case study. These results are useful to form further design strategies for urban furniture design concerning pandemic prevention and control.

From the decision model of ANP-QFD, three URs, namely, usability and easy access (UR1), sanitation and health (UR2) and emotional pleasure (UR3), are determined. There is no doubt that sanitation and health (UR2) is the most critical UR, receiving the highest weight of 0.589. During a pandemic situation, most people are inclined to focus on whether urban furniture can provide a safe and healthy guarantee for them. We should initially consider how to achieve sanitation and health requirements and propose design strategies based on these requirements in the practical design aspect. Usability and easy access (UR1) has a relative weight of 0.252, which is the second most important. Essentially, urban furniture is a kind of product in public places, providing social services for urban dwellers based on its function. Simultaneously, we cannot ignore emotional pleasure (UR3), reflecting users’ psychological requests and mental demands during pandemic situations. Although this requirement has the least weight among all URs, it still has a significant role in coping with pandemics. According to our understanding, the main reason for this may be that when people’s basic level requirements, such as health and safety are threatened, they will pay less attention to other higher-level requirements, like aesthetic requirement and self-achievement requirement, following the Maslow’s needs theory [71]. Therefore, during pandemic situations, we should give top priority to these fundamental requirements. 

On the other hand, seven DRs are put forward correspondingly. Smart system and technology (DR6) has the highest weight of 0.318, which is considered the most significant urban furniture design for pandemic prevention and control. This finding is consistent with previous studies. As Whitelaw stated, digital technologies are required for pandemics prevention and control during the process of surveillance, testing, contact tracing, and isolation [72]. More importantly, the strategies integrated with smart technology have been proven to be effective in some of the most successful countries during the COVID-19 pandemic. In Singapore, an application was developed to examine if two individuals are close to each other, based on Bluetooth technology in their smartphones [73]. Similarly, in South Korea, security camera footage, facial recognition technology and a global positioning system (GPS) were employed to provide real-time data for contact tracing [74]. The effective use of big data technology in Taiwan is a crucial cause of its low number of infected and deaths [25]. Hence, policymakers and designers should focus on strategies to combine urban furniture with smart technology for pandemic prevention and control. Besides, the other three DRs, including effective disinfection (DR2), ergonomics (DR5) and contact reduction (DR1) should also be focused on by designers and policymakers. This is because these elements share a relatively high level of importance, with weights of 0.181, 0.165 and 0.110, respectively. In addition to the relative weights of DRs, we should also focus on their inner dependency, which can demonstrate the association among them, thereby providing a reference for us to develop design strategies. From the value of W_4_, it can be derived that DR5 and DR6 impact DR1, while DR3 and DR4 have some associations with each other. These associations can be considered for further analysis when developing guidelines for pandemic prevention and control.

## 6. Strategies of Urban Furniture Design for Pandemic Prevention and Control

**Figure 8:**
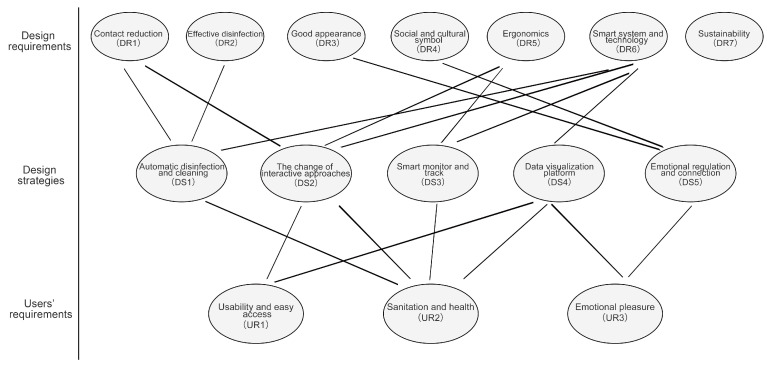
The links of design strategies with URs and DRs.

According to the relative weights of URs and DRs and their inner relationships, the design strategies of urban furniture design for pandemic prevention and control are proposed, guiding design practices from the functions, appearance, structure and interactive approaches of urban furniture; see Figure 8. It is worth noting that the links between design strategies and URs and DRs can demonstrate the results derived from the above sections. For example, the more links a DR has, the more critical it is.

### 6.1. Automatic Disinfection and Cleaning

This design strategy is concluded from UR2, DR1, DR2 and DR6. The function of automatic disinfection and cleaning can be added to urban furniture. As we know, urban dwellers have frequent access to urban furniture every day. In this case, with the increase in physical touch, viruses existing on urban furniture will see an increment, and the risk of contact transmission will grow. Hence, it is necessary to make the disinfection and cleaning of urban furniture automatic. One plan is to use automatic heating to substitute an original part of urban furniture, such as the surface of public tables and door handles. Generally, most viruses have fewer possibilities of survival under a high-temperature environment, so urban furniture with discontinuous heating can effectively kill viruses and prevent transmission. Similarly, other types of disinfection method can be considered, such as UV disinfection and filter disinfection. For example, we can install an Ultra-Violet (UV) device in the express cabinet, which can kill viruses on the express package, thereby avoiding the risk of person-to-object transmission. 

### 6.2. The Change of Interactive Approaches

This strategy concerns UR1, UR2, DR1, DR5 and DR6. Most contact transmission happens when people interact with urban furniture, so we can change the traditional interactive approaches between people and urban furniture. Taking the express cabinet as an example, during the current operating process, such as opening cabinets and typing numbers, there are many opportunities to touch the cabinet, which may increase the risk of transmission. Hence, voice control can be an alternative to the traditional keyboard control. Smartphones can also be employed as an interactive tool. Suppose we are going to complete the task of fetching an express. We can build links with the express cabinet system and then complete the task of inputting information and opening cabinets through remote control. Similarly, contactless screen techniques can be equipped in smart urban furniture, meaning that we can control the system through gestures at a close distance rather than real touch. Furthermore, facial recognition technology can be employed to open the gates in community entrances, which can prevent contact and track potential patients. In addition to these intelligent interactive methods, designers can also consider some mechanical structure that is simple to achieve. For instance, people often open trash bins through hand operation, but this interactive method can be substituted with foot operation. These contactless interactive methods, such as voice interaction, gesture interaction, physical interaction and intelligent terminal interaction, should be considered for integration with urban furniture for pandemic prevention and control.

### 6.3. Smart Monitor and Track

This design strategy is associated with UR2, DR5 and DR6. As close attachments in public spaces, urban furniture provides a platform involving entertainment, exercise, socialization and rest for citizens, in places where there is often a dense crowd. Therefore, urban furniture should function in terms of smart monitoring and tracking. For example, streetlights located at the entrance of communities can be equipped with a temperature sensor or a thermal imager, which can filter transient people with a high temperature. Using this, we can quickly trace and position suspicious patients, thereby preventing them from contaminating others. These smart sensors and imagers can also be installed on other urban furniture in communities which come into frequent contact with citizens, such as fitness facilities and bus stops. If all streetlights or signs in the key intersection in communities are installed with such smart monitor devices, they can comprise a tracking system, which can quickly locate suspicious groups and track their routes. To cope with COVID-19, people have installed such smart monitor devices in the entrance of public spaces, such as subway stations and airports.

### 6.4. Data Visualization Platform

This design strategy is related to UR1, UR2, UR3 and DR6. The absence of scientific recognition of pandemic-related knowledge may lead to a second-wave pandemic or a deteriorating situation. This is because many people do not know how to change their lifestyle and behaviors to adapt to pandemic situations. Simultaneously, some people may underestimate the robust destructiveness and infectivity of pandemics. Such issues are an obstacle to containing pandemic situations. Hence, launching effective campaigns to spread information about pandemics is crucial. In this case, one strategy is to build a data visualization platform that can demonstrate the real pandemic situation to the public. This platform can be set on a bulletin board, signs and bus stops in public spaces. As there are often some local residents gathering together in public areas, real-time information about pandemics can effectively warn these people and prevent such a massive assembly. Moreover, the data visualization platform can be integrated with a smart monitor and tracking system to show the physical status of community workers and residents, helping people better understand their surrounding pandemic situation, and thereby easing their negative mood. 

### 6.5. Emotional Regulation and Connection

This design strategy is associated with UR3, DR3 and DR4. According to Dr. Norman’s research, product design assumes a key role in regulating user emotions and building emotional connections, and this emotional connection can be reflected in the appearance of products [64]. Currently, people in many countries have experienced several waves of pandemics, which not only lead to an infectious disease, but also adversely affect people’s mental health. Although government agencies are trying their best to offer medical support for patients during the pandemic, we cannot ignore the mental health issues of these patients. In this case, urban furniture can also be designed to fulfill people’s emotional requirements and mitigate bad feelings. For example, the appearance of urban furniture can be designed to refer to “Pop art”, a fine art style developed in the early 1950s in the UK which expressed exaggeration, fun, and novelty, contrary to the cold and impersonal “realism style” [75]. Similarly, Scandinavian style can also be referred to, since this design style has the main characteristics of humanization and warmth, designed to cope with the cold and frozen weather conditions in Northern Europe [76]. A typical representative of Scandinavian-style is PH-lamps, using soft lighting and a round shape to express a humanized design [77]. Additionally, some other design styles, such as the childish style and gamification, can also be considered in the appearance design of urban furniture.

## 7. Conclusions

In modern society, pandemics have imposed grave threats to global health, public security and social order. Although humans can defeat pandemic situations through mass vaccination and the employment of antiviral drugs, we cannot stop the occurrence of the next pandemic. Hence, research concerning pandemic prevention and control is always a hot topic. In this article, we aim to cope with pandemic situations through urban furniture design, using a robust approach that integrates ANP with QFD. At the conceptual level, three user requirements, seven DRs and design strategies in five directions with respect to urban furniture design are proposed for pandemic prevention and control. These findings can contribute to the existing knowledge on how design fields can help cope with pandemics. Besides this, the effectiveness of the integrated ANP-QFD approach can be proven through two aspects. On the one hand, through the utilization of ANP-QFD, the priorities of URs and DRs and their interrelations can provide a more in-depth understanding of how to deploy them, thereby developing related design strategies to guide practical actions. Such a finding can answer the three questions put forward in the first section, indicating that the integrated ANP-QFD approach can allow researchers to gain a more comprehensive recognition of users’ expectations and the characteristics of products. This finding is consistent with the previous literature, which suggested that, when developing product design solutions, we should pay attention to factors that have a direct association with user expectations, and other factors that indirectly affect user satisfaction [41,78]. On the other hand, the integrated ANP-QFD method can overcome the vagueness and inaccuracy in the traditional ANP method, describing the user expectations and product features more systematically and validly. At the practical level, our research findings can provide a reference for government agencies, policymakers, designers and community managers, showcasing how to deal with pandemic situations in densely populated communities through design. Since there is a scarcity of research on coping with pandemics in the design fields, this article’s achievements can assist in providing a basis for related studies in this area.

However, there are still some limitations to our research. First, the scope of this study is restricted to densely populated communities in Hefei, China, which may not be transferred to other regions around the world. The main reason for this is that each nation may face rather diverse pandemic situations and have a wide variety of demographics, living circumstances, customs, lifestyles and economies, so the design strategies we propose in this article may not be suitable for other countries. Policymakers should consider their respective conditions and situations when dealing with pandemics. For instance, in our research, smart systems and technology (DR 6) were widely focused on by most respondents, indicating that this design requirement is supposed to play the most vital role in urban furniture design for pandemic prevention and control. However, we cannot ignore the fact that China has the second-largest economy, which can support the construction of urban infrastructure and facilities with intelligent technology. These costly technologies may not be available for all countries. In this case, we recommend that urban furniture with a simple structure and low cost is worth exploring by designers. Second, sustainability (DR7) received a relatively lower weight than other DRs, which was rarely explored in our research. However, we cannot ignore the crucial influence of this requirement. As mentioned above, the sustainability of a product is always a hot topic. In this article, people do not pay much attention to sustainability, as they want to find a quick and effective way to contain pandemics. However, some questions are worth exploring for researchers, such the sustainability of the urban furniture designed for pandemic prevention and control, whether the urban furniture designed for containing the current pandemics can be applied to another pandemic situation or some common infectious diseases, and whether there are other extensive uses for the redesigned urban furniture. Third, the data collection and analysis in our research rely heavily on respondents’ subjective justifications, which may be affected by their personal experience and understanding. Future studies are recommended, which can be incorporated with these experiments to enhance the accuracy and validity of the results.

## Figures and Tables

**Table 1:** Urban furniture in Chinese communities.

Type	Function	Image	Description
Express cabinet	Express service	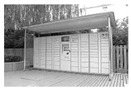	Offers express pick-up and drop-off services
Fitness facility	Exercise and entertainment	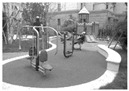	Provides a place where residents can exercise
Charging pile	Charging	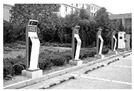	Provides energy for electrical vehicles and bicycles.
Seat	Rest	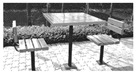	Includes places where several people can sit
Street light	Illumination	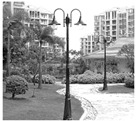	Offers light at night
Trash bin	Trash recycle	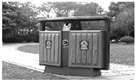	Holds rubbish until it is delivered
Sign	Guidance	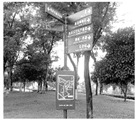	Offers directions and routes for citizens
Vending machine	Selling goods	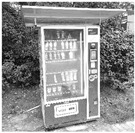	Sells snacks, beverages and cigarettes to customers automatically

**Table 2:** Description of respondents in the user interview.

Respondents	Description
Seven community workers	All of them have at least five years’ experience working in communities. They have an in-depth understanding of residents’ requirements. Furthermore, they have successfully organized some activities for pandemic prevention and control in the community.
Three community pharmacists	They are professional health workers in community clinics. All of them have undergone professional training, so they have professional knowledge of pandemic prevention and control.
Two PhD students in management	Both of them are skillful in community management and development. One has a PhD in community development, while the other is currently pursuing his PhD.
Two industrial designers	They are currently employed by professional design companies. Both of them have at least five years of working experience in industrial design and product design, with one in intermediate design and one as a senior designer.
Three university teachers in design	They are professional teachers in design fields. All of them have at least 15-years’ teaching and research experience in design fields, with two being associate professors and one a professor.
Three PhD students in design	All of them are currently pursuing their PhD in design.
Two technicians	Both of them have at least five years of working experience in manufacturing and maintaining urban furniture, and they are familiar with the manufacturing of urban furniture.

**Table 3:** Demographic information and partial contents of the questionnaire survey.

Attribute	Value	Frequency	Attribute	Value	Frequency
Gender	Male	76	Attitudes toward the roles of existing urban furniture in pandemic prevention and control	Very useless	42
	Female	88		Useless	97
Age	21–30	29		Slight useful	23
	31–40	46		Useful	2
	41–50	49		Very useful	0
	Above 50	40	Attitudes toward the associations between urban furniture design and pandemic prevention and control	Very weak	3
Occupation	Local residents	118		Weak	12
	Community workers	28		General	38
	Pharmacists	5		Strong	84
	Designers	13		Very strong	27

**Table 4:** User requirements (URs) of urban furniture design for pandemic prevention and control.

Notation	User Requirement	Description
UR1	Usability and easy access	Provide safe, effective and efficient using conditions for users
UR2	Sanitation and health	Exposure to a clean circumstance where there is no virus and contamination
UR3	Emotional pleasure	Evoke positive emotions and ease users’ negative feelings when users are interacting with a product

**Table 5:** Design requirements (DRs) of urban furniture design for pandemic prevention and control.

Notation	Design Requirement	Description
DR1	Contact reduction	Reduce the frequency of contact to lower risk of transmission and help users keep a considerable physical distance from each other
DR2	Effective disinfection	Eliminate viruses existing on objects through various disinfection methods
DR3	Good appearance	Improve users’ emotions and mitigate their bad feelings through suitable form, bright colors and the right proportions
DR4	Social and cultural symbol	Have a sense of cultural identity and a sense of belonging to a specific culture
DR5	Ergonomics	Easy access to completing a task, offer a sense of accomplishment and a satisfying experience, and cater to ergonomic principles
DR6	Smart system and technology	Incorporate features of sensor, actuation, and control based on intelligent techniques and system
DR7	Sustainability	Environmentally friendly design that can minimize the harmful impact on our surroundings

**Table 6:** The importance of levels 1–9 in the questionnaire.

Level	Importance	Implication
1	Equivalently important	One matrix is similarly important as the other
3	Slightly important	One matrix is slightly more important than the other
5	Moderately important	One matrix is moderately more important than the other
7	Considerably important	One matrix is considerably more important than the other
9	Dramatically important	One matrix is dramatically more important than the other
2, 4, 6, 8	------	Mediating degrees between these mentioned above

**Table 7:** Relative weights of each UR (W_1_).

W_1_	UR1	UR2	UR3	Weight
UR1	1	1/3	2	0.252
UR2	3	1	3	0.589
UR3	1/2	1/3	1	0.159

Note: W_1_ is an eigenvector; UR1 = Usability and easy access; UR2 = Sanitation and health; UR3 = Emotional pleasure.

**Table 8:** Relative weights of DRs with respect to UR1 (W_2_).

W2-UR1	DR1	DR2	DR3	DR4	DR5	DR6	DR7	Weight
DR1	1	1/6	2	2	1/7	1/5	1/2	0.052
DR2	6	1	6	6	1/2	2	4	0.256
DR3	1/2	1/6	1	1	1/7	1/5	1/4	0.035
DR4	1/2	1/6	1	1	1/7	1/6	1/4	0.034
DR5	7	2	7	7	1	2	4	0.334
DR6	5	1/2	5	6	1/2	1	3	0.192
DR7	2	1/4	4	4	1/4	1/3	1	0.097

Note: W_2_ is a correlation matrix; UR1 = Usability and easy access; DR1 = Contact reduction; DR2 = Effective disinfection; DR3 = Emotional pleasure; DR4 = Social and cultural symbol; DR5 = Ergonomics; DR6 = Smart system and technology; DR7 = Sustainability.

**Table 9:** Relative weights of DRs with respect to each UR (W_2_).

W_2_	UR1	UR2	UR3
DR1	0.052	0.212	0.039
DR2	0.256	0.212	0.061
DR3	0.035	0.028	0.266
DR4	0.034	0.048	0.164
DR5	0.334	0.103	0.099
DR6	0.192	0.327	0.099
DR7	0.097	0.070	0.272

Note: W_2_ is a correlation matrix; UR1 = Usability and easy access; UR2 = Sanitation and health; UR3 = Emotional pleasure; DR1 = Contact reduction; DR2 = Effective disinfection; DR3 = Emotional pleasure; DR4 = Social and cultural symbol; DR5 = Ergonomics; DR6 = Smart system and technology; DR7 = Sustainability.

**Table 10:** Relative weights of URs with respect to each UR (W_3_).

W_3_	UR1	UR2	UR3
UR1	0.681	0.284	0.123
UR2	0.201	0.619	0.320
UR3	0.118	0.097	0.557

Note: W_3_ is a correlation matrix; UR1 = Usability and easy access; UR2 = Sanitation and health; UR3 = Emotional pleasure.

**Table 11:** Relative weights of DRs with respect to each DR (W_4_).

W_4_	DR1	DR2	DR3	DR4	DR5	DR6	DR7
DR1	0.557	0.000	0.000	0.000	0.116	0.080	0.000
DR2	0.000	0.667	0.000	0.000	0.074	0.139	0.000
DR3	0.000	0.000	0.539	0.400	0.000	0.000	0.122
DR4	0.000	0.000	0.297	0.400	0.000	0.000	0.122
DR5	0.123	0.000	0.000	0.000	0.405	0.315	0.000
DR6	0.320	0.333	0.000	0.000	0.405	0.466	0.220
DR7	0.000	0.000	0.164	0.200	0.000	0.000	0.536

Note: W_4_ is a correlation matrix; DR1 = Contact reduction; DR2 = Effective disinfection; DR3 = Emotional pleasure; DR4 = Social and cultural symbol; DR5 = Ergonomics; DR6 = Smart system and technology; DR7 = Sustainability.

**Table 12:** Rankings of DRs in the traditional QFD and the integrated ANP-QFD.

Rankings	The traditional QFD	The Integrated ANP-QFD
DR1	5	4
DR2	3	2
DR3	6	6
DR4	7	7
DR5	2	3
DR6	1	1
DR7	4	5

Note: DR1 = Contact reduction; DR2 = Effective disinfection; DR3 = Emotional pleasure; DR4 = Social and cultural symbol; DR5 = Ergonomics; DR6 = Smart system and technology; DR7 = Sustainability.

## Data Availability

The data presented in this study are available on request from the corresponding author.

## Appendix A

**Table A1:** Calculating relative weights of DRs in terms of UR2 through pairwise comparison to determine W2.

W_2_-UR2	DR1	DR2	DR3	DR4	DR5	DR6	DR7	Weight
DR1	1	1	6	5	3	1/2	4	0.212
DR2	1	1	6	5	3	1/2	4	0.212
DR3	1/6	1/6	1	1/3	1/5	1/7	1/4	0.028
DR4	1/5	1/5	3	1	1/3	1/6	1/2	0.048
DR5	1/3	1/3	5	3	1	1/4	2	0.103
DR6	2	2	7	6	4	1	5	0.327
DR7	1/4	1/4	4	2	1/2	1/5	1	0.070

**Table A2:** Calculating relative weights of DRs in terms of UR3 through pairwise comparison to determine W2.

W_2_-UR3	DR1	DR2	DR3	DR4	DR5	DR6	DR7	Weight
DR1	1	1/2	1/5	1/4	1/3	1/3	1/6	0.039
DR2	2	1	1/4	1/3	1/2	1/2	1/4	0.061
DR3	5	4	1	2	3	3	1	0.266
DR4	4	3	1/2	1	2	2	1/2	0.164
DR5	3	2	1/3	1/2	1	1	1/3	0.099
DR6	3	2	1/3	1/2	1	1	1/3	0.099
DR7	6	4	1	2	3	3	1	0.272

**Table A3:** Calculating relative weights of URs interms of UR1 through pairwise comparison to determine W3.

W_3_-UR1	UR1	UR2	UR3	Weight
UR1	1	4	5	0.681
UR2	1/4	1	2	0.201
UR3	1/5	1/2	1	0.118

**Table A4:** Calculating relative weights of URs interms of UR2 through pairwise comparison to determine W3.

W_3_-UR2	UR1	UR2	UR3	Weight
UR1	1	1/3	4	0.284
UR2	3	1	5	0.619
UR3	1/4	1/5	1	0.097

**Table A5:** Calculating relative weights of URs interms of UR3 through pairwise comparison to determine W3.

W_3_-UR3	UR1	UR2	UR3	Weight
UR1	1	1/3	1/4	0.123
UR2	3	1	1/2	0.320
UR3	4	2	1	0.557
